# 7^th^ Brazilian Guideline of Arterial Hypertension: Chapter 7 - Pharmacological Treatment

**DOI:** 10.5935/abc.20160157

**Published:** 2016-09

**Authors:** MVB Malachias, PCV Paulo César Veiga Jardim, FA Almeida, E Lima Júnior, GS Feitosa

## Objectives

The treatment of AH is ultimately aimed at reducing CV morbidity and mortality.^1-11^ Clinical studies of outcome have provided scientific evidence of the benefits of the use of diuretics (DIUs) (GR: I; LE: A),^5,10-15^ beta-blockers (BBs) (GR: I; LE: A),^10-13,16^ calcium-channel blockers (CCBs) (GR: I; LE: A),^10,11,15,17-23^ angiotensin-converting-enzyme inhibitors (ACEIs) (GR: I; LE: A)^10,11,15,17,18,24-26^ and angiotensin-receptor blockers (ARBs) (GR: I; LE: A).^10,11,27-33^ It is worth noting that most of those studies have used an association of drugs. Based on the information available, the protection observed does not depend on the type of drug used, but mainly on BP reduction.^7,9-11,34^ Recent meta-analyses have reported that the benefits obtained from BB are smaller^10,11,35-37^ as compared to those provided by the other drug groups, and, thus, BBs should be reserved for specific situations. Regarding alpha-blockers and direct vasodilators, there is no effective information on the outcomes of morbidity and mortality. Regarding direct renin inhibitors, only one study of outcome in diabetic patients has been early interrupted due to lack of benefits and possible harm.^38^ The higher the CV risk, the greater the benefits, which occur even for small BP elevations.^3-6,8,9,39^

### General principles of the pharmacological treatment

When pharmacological treatment is indicated, the patient should be instructed about the importance of its continuity, the occasional need for dose adjustment and change or association of drugs, and the occasional appearance of adverse effects.

For one medicine to be indicated, it should preferably:

- have shown the ability to reduce CV morbidity and mortality;
- be effective orally;
- be well tolerated;
- be taken the fewest possible times per day;
- be started at the smallest effective doses;
- be able to be used in association;
- be used for at least four weeks, before any change, except for special situations;
- have quality control in its production.

### Choice of the medication

All antihypertensive drugs available can be used if specific indications and contraindications are observed (Table 1). The initial preference is always for those with confirmed action in decreasing CV events, being the others reserved for special cases that require the association of multiple drugs to achieve BP targets.

**Table 1:** Antihypertensive drugs available

- DIUs (GR: I; LE: A)
- adrenergic inhibitors
- Central action – central alpha-2 agonists (GR: IIb; LE: C)
- BBs – beta adrenergic blockers (GR: I; LE: A)
- Alpha-blockers – alpha-1 adrenergic blockers (GR: IIb; LE: C)
- Direct vasodilators (GR: IIb; LE: C)
- CCBs (GR: I; LE: A)
- ACEIs (GR: I; LE: A)
- ARBs (GR: I; LE: A)
- Direct renin inhibitors (GR: IIb; LE: C)

## General characteristics of antihypertensive drugs

### Diuretics

The mechanisms of antihypertensive action of DIUs are initially related to their natriuretic effects, with a decrease in the extracellular volume. After 4-6 weeks, the circulating volume normalizes and a reduction in peripheral vascular resistance (PVR) occurs. Diuretics reduce BP and CV morbidity and mortality.^12,14,15^ Their antihypertensive effect is not directly related to their doses, but the side effects are.

Thiazide or similar DIUs (chlorthalidone, hydrochlorothiazide and indapamide) at low doses should be preferred, because they are milder and have a longer time of action. Loop DIUs (furosemide and bumetanide) should be reserved for cases of renal failure (creatinine > 2.0 mg/dL or estimated GFR < 30 mL/min/1.73m^2^) and edema (HF or renal failure). Potassium-sparing DIUs (spironolactone and amiloride) are usually associated with a thiazide or loop DIU.

#### Adverse effects

Their major adverse effects are weakness, cramps, hypovolemia and erectile dysfunction. From the metabolic viewpoint, hypopotassemia is the most common, occasionally accompanied by hypomagnesemia, which can induce ventricular arrhythmias, mainly extrasystole. Diuretics can cause glucose intolerance by reducing insulin release, increasing the risk for type 2 DM. Uric acid increase is an almost universal effect of DIUs, of undocumented clinical consequences, except for triggering gout crises in predisposed individuals. The use of low doses decreases the risk for adverse effects, without hindering the antihypertensive efficacy, especially when associated with other drug classes. Spironolactone can cause hyperpotassemia, particularly in patients with impaired renal function.

### Central action agents

Alpha-agonists of central action stimulate alpha-2 receptors involved in sympatho-inhibitory mechanisms.^40^ Not all alpha-agonists of central action are selective. Their well-defined effects are as follows: a decrease in sympathetic activity and reflex of baroreceptors, contributing to relative bradycardia and postural hypotension; mild decrease in PVR and cardiac output; a reduction in serum levels of renin; and fluid retention.

Some representatives of that group are: methyldopa, clonidine, guanabenz and inhibitors of imidazoline receptors (moxonidine and rilmenidine).^41^

Clonidine can be useful in hypertensive situations associated with: restless legs syndrome,^42^ withdrawal of opioids,^43^ menopausal hot flushes,^44^ diarrhea associated with diabetic neuropathy,^45^ and sympathetic hyperactivity of patients with alcoholic cirrosis.^46^ These drugs have no unwanted metabolic effect, because they interfere with neither peripheral resistance to insulin nor lipid profile.

#### Adverse effects

Methyldopa can cause autoimmune reactions, such as fever, hemolytic anemia, galactorrhea and liver dysfunction, which, in most cases, disappear with use cessation. If an adverse reaction occurs, it can be replaced by another central alpha-agonist.^41^ Clonidine has a higher risk for the rebound effect with discontinuation, especially when associated with a BB, and can be dangerous in the preoperative period.^40^ The drugs in this class have adverse reactions due to their central action, such as drowsiness, sedation, dry mouth, fatigue, postural hypotension, and erectile dysfunction.^40,41^

### Beta-blockers

Beta-blockers promote initial decrease in cardiac output and renin secretion, with readaptation of baroreceptors and decrease in catecholamines in nervous synapses.^47,48^ In addition to such actions, third-generation drugs (carvedilol, nebivolol) have a vasodilating effect via different mechanisms: carvedilol, via concomitant blockade of alpha-1 adrenergic receptor;^47-50^ and nebivolol, by increasing nitric oxide synthesis and release on the vascular endothelium.^47,48,50^ Propranolol is useful to patients with essential tremor, hyperkinetic syndromes, vascular headache and portal hypertension.^47,48^

#### Adverse effects

They consist of bronchospasm, bradycardia, atrioventricular conduction disorders, peripheral vasoconstriction, insomnia, nightmares, psychic depression, asthenia and sexual dysfunction. First- and second-generation BBs are formally contraindicated to patients with bronchial asthma, chronic obstructive pulmonary disease (COPD) and second- and third-degree atrioventricular blocks. They can cause glucose intolerance, induce new cases of DM, and lead to hypertriglyceridemia with LDL-cholesterol elevation and HDL-cholesterol reduction. The impact on glucose metabolism is potentiated when combined with DIUs. Third-generation BBs (carvedilol and nebivolol) have neutral impact or can even improve the glucose and lipid metabolism, possibly because of the vasodilating effect with decrease in insulin resistance and improvement of glucose uptake by peripheral tissues.^47,50^ Studies on nebivolol have shown less sexual dysfunction, possibly because of the effect on endothelial nitric oxide synthesis.^47,50^

### Alpha-blockers

Alpha-blockers act as competitive antagonists of postsynaptic alpha-1 receptors, leading to a reduction in PVR without major changes in cardiac output.^41^ Some representatives of this drug class are doxazosin, prazosin and terazosin. The hypotensive effect is mild in monotherapy, the combined use being preferred. They have a favorable and discrete action on the lipid and glucose metabolisms, especially improving the symptoms related to benign prostate hypertrophy.^41^

#### Adverse effects

Alpha-blockers can cause symptomatic hypotension on the first dose. The phenomenon of tolerance is frequent, requiring increasing doses. Women can have urine incontinence. There is evidence that patients treated with doxazosin are at higher risk for CHF.^41^

### Direct acting vasodilators

Representatives of this drug class are hydralazine and minoxidil. They act directly, relaxing arterial smooth muscle, leading to a PVR reduction.^40^

#### Adverse effects

The side effects of hydralazine are headache, flushing, reflex tachycardia and lupus-like reaction (dose-dependent).^41^ Hydralazine should be used carefully in patients with CAD, and avoided in those with dissecting aortic aneurysm and a recent cerebral hemorrhage episode. In addition, it can cause anorexia, nausea, vomiting and diarrhea. A common side effect of minoxidil is hirsutism, in approximately 80% of the patients. A less common side effect is the general expansion of the circulating volume and reflex tachycardia.

### Calcium-channel blockers

Calcium channel blockers cause a reduction in PVR, because of the decreased calcium amount inside arteriolar smooth muscle cells, due to calcium channel blockade in their membranes.^51^ They are classified as dihydropyridine and nondihydropyridine CCBs.

Dihydropyridine CCBs (amlodipine, nifedipine, felodipine, nitrendipine, manidipine, lercanidipine, levamlodipine, lacidipine, isradipine, nisoldipine, nimodipine) have mainly a vasodilating effect, with minimum interference in HR and systolic function, being, thus, more often used as antihypertensive agents. Nondihydropyridine CCBs, such as phenylalkylamines (verapamil) and benzothiazepines (diltiazem), have a lower vasodilating effect, can cause bradycardia and have an antiarrhythmic effect, which limit their use to specific cases. Nondihydropyridine CCBs can depress the systolic function, mainly in patients with systolic dysfunction prior to their use, and, thus, should be avoided in that condition. Long-acting CCBs should be preferred to prevent unwanted oscillations in HR and BP. They are effective antihypertensive drugs that reduce CV morbidity and mortality.^52-55^ A study of outcome has reassured the efficacy, tolerability and safety of this drug class for the AH treatment of patients with CAD,^56^ being an alternative when BBs cannot be used, or even in association, in cases of refractory angina.

#### Adverse effects

Ankle swelling is usually the most common side effect, resulting from the vasodilating action (more arterial than venous), which causes capillary transudation. Throbbing headache and dizziness are not uncommon. Facial blushing is more common with fast-acting dihydropyridine CCBs. Hyperchromia of the distal third of the legs (ochre dermatitis) and gingival hypertrophy might occur. Such effects can be dose-dependent. Verapamil and diltiazem can worsen HF, bradycardia and atrioventricular block. Constipation is observed with verapamil.^55^

### Angiotensin-converting-enzyme inhibitors

Angiotensin-converting-enzyme inhibitors are effective antihypertensive drugs whose major action is inhibition of angiotensin-converting-enzyme, hindering transformation of angiotensin I into angiotensin II, a vasoconstrictor. They are effective to treat AH, reducing CV morbidity and mortality.^57^ They are useful in many other CV conditions, such as HF with reduced ejection fraction, post-infarction anti-remodeling, and might have antiatherosclerotic properties. They delay renal function decline in patients with diabetic nephropathy or nephropathy of other etiologies.^58^

#### Adverse effects

Usually well-tolerated by most hypertensive patients, dry cough is their major side effect, affecting 5-20% of patients. Angioneurotic edema^59^ and skin rash are rare. Serum urea and creatinine elevation, usually small and reversible, is a transient phenomenon observed in the initial use of ACEIs in patients with renal failure.^60^ In the long run, ACEIs are effective to halt the progression of CKD. They can cause hyperpotassemia in patients with renal failure, mainly those with DM. They can reduce GFR and increase the levels of urea, creatinine and potassium in patients with bilateral stenosis of the renal arteries or renal artery stenosis in a single functioning kidney. They are contraindicated during pegnancy,^61^ because of the risk of fetal complicactions.^62^ Thus, they should be carefully used and often monitored in adolescents and childbearing-age women.

### Angiotensin II AT1 receptor blockers

The ARBs antagonize the action of angiotensin II via the specific blockade of AT1 receptors, responsible for angiotensin II own actions of vasoconstriction, proliferation and stimulation of aldosterone release. In the AH treatment, especially of populations at high CV risk or with comorbidities, ARBs reduce CV and renal (diabetic nephropathy) morbidity and mortality.^27-29,63-66^

#### Adverse effects

Adverse effects related to ARBs are not common, exanthema being rarely observed. Similarly to ACEIs, ARBs are contraindicated during pregnancy, and the same care should be taken for childbearing-age women.

### Direct renin inhibitors

Aliskiren, the only representative of this drug class available for clinical use, causes direct renin inhibition with consequent decrease in angiotensin II production.^67^ Other actions might contribute to BP lowering and tissue protection, such as the reduction in renin plasma activity,^67^ the blockade of a renin/prorenin receptor,^68^ and the decrease in intracellular angiotensin II production.^69^ Studies of antihypertensive efficacy have confirmed its ability in monotherapy to lower BP in an intensity similar to that of other antihypertensive drugs.^70^ There is, however, no evidence of its benefits on morbidity and mortality.

#### Adverse effects

They are well tolerated. Skin rash, diarrhea [especially at high doses (> 300 mg/day)], CPK increase, and cough are the most frequent events, whose incidence is usually < 1%. Their use is contraindicated during pregnancy.

### The beginning of pharmacological treatment

Pharmacological treatment is indicated for individuals with stage 1 AH and at low and intermediate CV risk, when nonpharmacological measures proved ineffective after an initial period of at least 90 days. In especial situations, in which access and/or return to medical care is difficult, the initial use of antihypertensive drugs, even for that group of patients, might be considered. For individuals with stage 1 AH and at high CV risk or with established CVD, the use of antihypertensive agents should be started immediately. Likewise, for patients with stage 2 and 3 AH, regardless of the CV risk, pharmacological treatment should be started immediately. For prehypertensive individuals, pharmacological treatment might be an option, considering the CV risk and/or presence of CVD. For 60- to 79-year-old patients with SBP ≥ 140 mm Hg and those ≥ 80 years with SBP ≥ 160 mm Hg, pharmacological therapy should begin earlier.

### Therapeutic schemes

**Figure 1:**
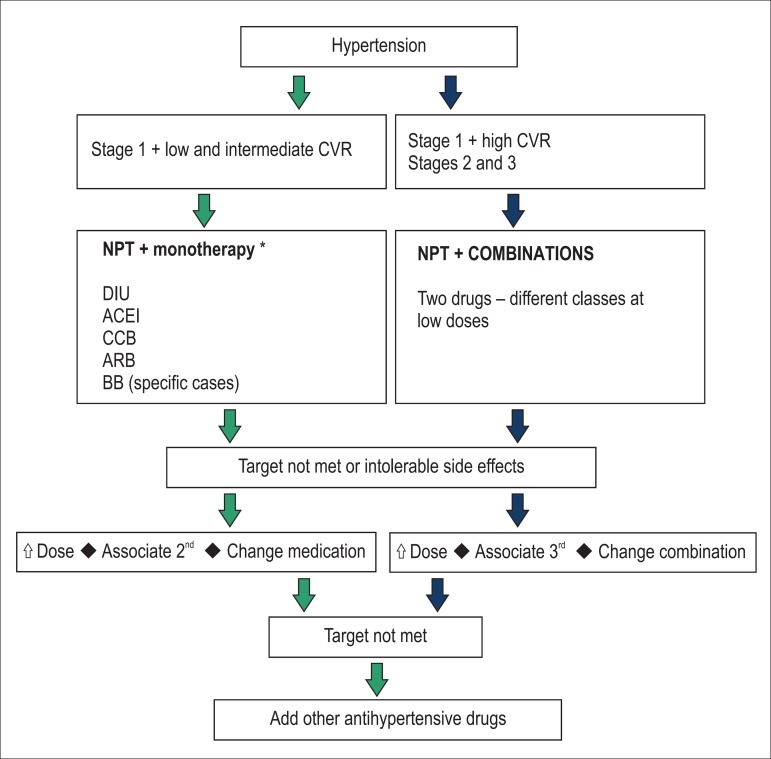
Flowchart for the treatment of hypertension. CVR: cardiovascular risk; NPT: non-pharmacological treatment; DIU: diuretics; ACEI: angiotensin-convertingenzyme inhibitors; CCB: calcium-channel blockers; ARB: angiotensin-receptor blockers; BB: beta-blockers.

The pharmacological treatment can be performed with one or more drug classes, as required, to meet the BP targets and according to specific situations (Figure 1).

### Monotherapy

Monotherapy can be the initial antihypertensive strategy for stage 1 AH patients at low and intermediate CV risk. However, depending on the BP target to be achieved, most patients will require drug combination. The treatment should be individualized, and the initial choice of drug to be used as monotherapy should be based on the following aspects: ability to lower CV morbidity and mortality; predominant pathophysiological mechanism in the patient to be treated; individual characteristics; associated diseases; and socioeconomic conditions.

**Figure 2:**
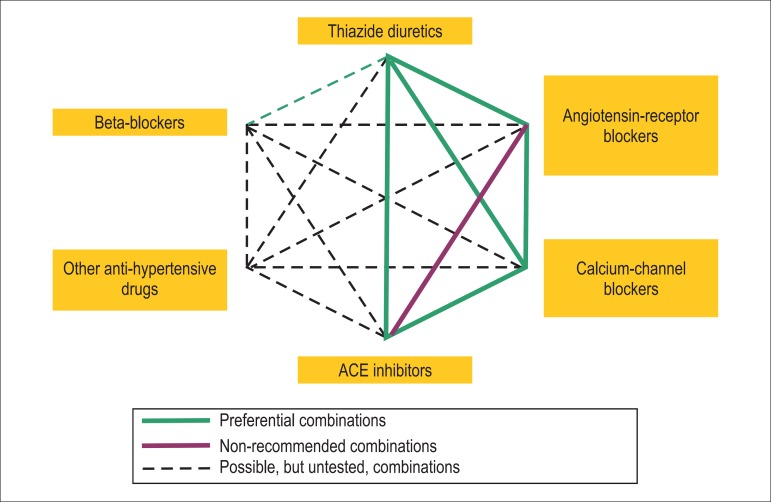
Preferential associations of drugs according to mechanisms of action and synergy. Adapted from Journal of Hypertension 2007, 25:1751-1762.

Based on those criteria, the classes of antihypertensive drugs currently preferred for BP control in the initial monotherapy are as follows (Figures 1 and 2):

- Thiazide DIUs (preference for chlorthalidone);^5,10-15,39,71,72^
- ACEIs;^7-11,15,17,18,24-26^
- CCBs;^7-11,15,17-23^
- ARBs.^10,11,27-33,73-78^

It is worth noting that DIUs have the greatest evidence of effectiveness regarding CV outcomes, with clear benefits for all types of events. In some situations, the indication of a certain group is reinforced, depending on the existing comorbidity. A BB can be considered the initial drug in certain situations, such as the presence of supraventricular arrhythmias, migraine, HF and CAD, and, in the last two conditions, the BB should be associated with other drugs.^47,48^

The dosage should be adjusted to provide BP lowering to levels considered adequate for each case (therapeutic targets).^1,2,8,79^ If the therapeutic objective is not achieved with the initial monotherapy, there are three possible options:

- If the result is partial, but with no adverse effect, the dose of the drug used should be increased, and association with an antihypertensive drug of another group should be considered;
- When the therapeutic effect expected at the maximum dose recommended is not obtained or in the presence of adverse events, the following is recommended: replace the antihypertensive agent initially used, reduce its dosage, and add another antihypertensive agent of a different class or use another association of drugs;
- If the response is inappropriate, three or more drugs should be associated (Figure 1).

### Combination of drugs

Most patients will need more than one drug to achieve BP targets. Therefore, patients with stage 1 AH and at high or very high CV risk or with CVD associated and those with stage 2 or 3 AH with or without other CVRF associated should be considered for drug combination (Figure 1). In addition, the association of two drugs at low doses for stage 1 hypertensive patients, even at low or intermediate CV risk, although not preferential, can be considered.

When choosing the drugs to be combined, antihypertensive agents sharing the same mechanism of action should be avoided, except for the association of thiazide DIUs and potassium-sparing DIUs. Loop DIUs should be reserved for individuals with GFR < 30 mL/min or severe edema. Associations with synergistic action provide better results (Figure 2).^80^

### Particularities of the associations

Less tested associations should be reserved for cases requiring a larger number of drugs;

The association of BB and DIU should be performed carefully for patients with glucose metabolism changes, because both drugs contribute to worsen them;

The association of ACEI and ARB is not recommended, because, in addition to showing no benefit in CV outcomes, it increases the risk for adverse effects;^33^

Studies comparing directly the associations are scarce. A study has shown that the combination of ACEI and CCB, as compared to the association of ACEI and DIU, was more effective in lowering CV morbidity and mortality and the progression of kidney disease, for a similar reduction in BP, mainly in non-obese individuals.^81,82^

Combinations can be performed freely with separate drugs or in a fixed association (same galenic formulation). If, on the one hand, free combinations allow us to choose the dose of each component, on the other hand, the use of fixed associations favors adherence to treatment, because of the smaller number of tablets.^83^

If BP control is not attained with two drugs, some decisions can be made:

- in case of partial result and no side effect, the dose of the initial combination can be increased, or one more antihypertensive agent of another drug class can be added;
- when the target is not achieved at the maximum dose recommended, or if adverse events occur, the combination should be replaced;
- if, at maximum doses, BP control is not attained, other antihypertensive drugs should be associated (Figure 1).

If a DIU was not the first choice and is not being used in the association of two drugs, it should be the third drug to be added. Its use potentiates the antihypertensive action of any initial drug.

In cases of resistant AH (lack of BP control with at least three drugs at their maximum doses tolerated, one being a DIU), association of spironolactone is indicated.^84-86^ Sympatholytic drugs of central action (clonidine) or BBs can be an alternative to the fourth drug to be added, direct vasodilators being reserved for special cases and in association with a DIU and a BB.
